# Hepatitis B Virus Immunopathology, Model Systems, and Current Therapies

**DOI:** 10.3389/fimmu.2017.00436

**Published:** 2017-04-13

**Authors:** Praneet Sandhu, Mohammad Haque, Tessa Humphries-Bickley, Swetha Ravi, Jianxun Song

**Affiliations:** ^1^Department of Microbiology and Immunology, The Pennsylvania State University College of Medicine, Hershey, PA, USA

**Keywords:** hepatitis B virus, immunopathology, model systems, therapies, immunotherapy for HBV

## Abstract

Most people develop acute hepatitis B virus (HBV)-related hepatitis that is controlled by both humoral and cellular immune responses following acute infection. However, a number of individuals in HBV-endemic areas fail to resolve the infection and consequently become chronic carriers. While a vaccine is available and new antiviral drugs are being developed, elimination of persistently infected cells is still a major issue. Standard treatment in HBV infection includes IFN-α, nucleoside, or nucleotide analogs, which has direct antiviral activity and immune modulatory capacities. However, immunological control of the virus is often not durable. A robust T-cell response is associated with control of HBV infection and liver damage; however, HBV-specific T cells are deleted, dysfunctional, or become exhausted in chronic hepatitis patients. As a result, efforts to restore virus-specific T-cell immunity in chronic HBV patients using antiviral therapy, immunomodulatory cytokines, or therapeutic vaccination have had little success. Adoptive cell transfer of T cells with specificity for HBV antigen^+^ cells represents an approach aiming to ultimately eliminate residual hepatocytes carrying HBV covalently closed circular DNA (cccDNA). Here, we discuss recent findings describing HBV immunopathology, model systems, and current therapies.

## Introduction

Hepatitis B virus (HBV) belongs to the *Orthohepadna* genus of the *Hepadnaviridae* family of virus and has a unique replication strategy wherein it replicates its 3.2 kb DNA genome using an RNA intermediate *via* reverse transcription (1). HBV infects hepatocytes to cause pathology in the liver as an acute or chronic infection. Acute infection HBV was classified by a physician, Dr. MacCullum, to be responsible for causing serum hepatitis in 1947 transmitted through blood (2). However, the discovery of a virus-associated antigen occurred serendipitously in an Australian aboriginal patient while studying polymorphisms in proteins and was called Australia antigen by Dr. Baruch S. Blumberg in 1965 (2–5). In 1970, the complete infectious virus was described by Dr. D. S. Dane using electron microscopy (6). The virus completes its lifecycle in the host hepatocyte and its tropism is limited to humans, chimpanzees, and tupaia (tree shrew) (7). Thus, it has been very hard to model this disease in animals other than chimpanzees, which recapitulates the disease most closely to humans.

Hepatitis B virus causes acute or chronic infection in humans with long and variable incubation times ranging from 8 weeks to 6 months (8). Acute infection can be characterized by presence of surface antigen of HBV (HBsAg), secreted viral protein (HBeAg), and alanine and aspartate aminotransferase in serum (9). Following this, there is appearance of antibodies against core antigen of HBV (HBcAg) followed by HBeAg and HBsAg in serum, which aids in recovery of patient and clearance HBV infection (9). Acute infection remains asymptomatic for many patients while others experience symptoms such as nausea, hepatitis (10). Chronic infection develops similar to acute infection but the patient does not recover from HBV infection as high levels of HBV DNA and HBsAg in serum persist long after exposure to HBV (11). The risk of chronic HBV infection is highest for infants infected perinatally and older people (12).

The lifecycle of HBV begins with the virus binding to its receptor on the hepatocyte surface. This receptor remained undefined for HBV for a long time but recently it has been observed that sodium taurocholate cotransporting polypeptide (NTCP) acts as one of the receptors for HBV (13). However, expression of human NTCP in HBV non-permissive mouse cell lines does not confer permissivity to such cells, suggesting that there may be other coreceptors required for HBV entry or lack of host factors required for HBV replication within mouse cells (14). Post-entry, the nucleocapsid containing relaxed, circular DNA (rcDNA) is trafficked from the cytoplasm to the nucleus; where rcDNA is released from nucleocapsid at nuclear pore and undergoes conversion to covalently closed circular DNA (cccDNA) *via* DNA repair mechanisms of the host (15, 16). This cccDNA is responsible for establishment of chronic HBV disease as cccDNA acquires host histone proteins to form a mini-chromosome structure to support low-level HBV replication. HBV transcribes for (a) core nucleocapsid protein and pre-core protein from core/pre-core transcript; (b) large (L), middle (M), and small (S) proteins from the preS1- and preS2-RNA transcript; (c) reverse transcriptase and polymerase (Pol)-associated proteins from Pol transcript; and (d) multi-functional protein X (17). The cccDNA is transcribed to these four transcripts that are translated in the cytoplasm and assemble the nucleocapsid containing pre-genomic RNA (pgRNA) (18). This pgRNA synthesizes positive and negative strand of the DNA to assemble viral particles containing rcDNA that acquire lipid membranes through the Golgi complex and bud out from the host cell surface (17). Additionally, these nucleocapsids can re-enter the nucleus prior to membrane acquisition and enter the HBV replication process as rcDNA (17). During this process, small (S) protein (filaments and spheres) and pre-core transcript are also shed in large numbers from the hepatocyte (19). Thus, HBV follows this complex lifecycle involving reverse transcription similar to retro-viruses, unlike DNA viruses.

## Immune Pathogenesis of HBV

### Cytosolic/Toll-Like Receptor (TLR) Sensing of HBV

Innate sensing of viruses can occur through TLRs and cytosolic sensors that recognize viral DNA and RNA. MDA-5 and RIG-I, RNA cytosolic sensors, have been shown to respond to HBV infection. Loss of MDA-5 in Huh7 cells and C57BL/6 mice caused increased HBV replication but did not result in robust IFN-β induction upon overexpression of MDA-5 in Huh7 cells transfected with HBV genome (20). Recent studies reported that RIG-I recognized the 5′ epsilon structure of HBV pgRNA and caused induction of IFN-λ in HBV-infected primary human hepatocytes (PHHs) and humanized mice (21). However, both studies used different genotypes of HBV, which may explain the conflicting results reported. There is also evidence to suggest that TLRs might have a role in clearance of HBV with activation of TLR signaling ramping up the interferon α and β (IFN-α/β) production (22). The direct ligand that is sensed by TLR during HBV infection is not yet known. In addition, *in vitro* studies in hepatoma-derived cell lines also show that HBV proteins Pol and X target DNA and RNA sensing adaptor proteins stimulator of interferon genes and mitochondrial antiviral signaling, respectively (23, 24). HBV Pol was also shown to interfere with downstream signaling of cytosolic sensing by blocking tank-binding kinase 1 (25). This suggests that HBV can activate different cytosolic sensors but has robust evasion strategies to combat cellular defense response and inhibit interferon production. However, most studies utilized methods of overexpression of cytosolic sensor proteins in cell lines that are defective for innate cytosolic sensing. Thus, mechanisms that contribute to HBV detection by cytosolic sensors and subsequent evasion by the virus during early stage of pathogenesis are still not completely understood.

### Immune Response against HBV

Hepatitis B virus engages different immune components over time as it progresses through its pathogenesis. It is primarily a concerted action of interferon-γ (IFN-γ) and cytolytic CD8^+^ T cells that target infected hepatocytes during acute phase of infection (26). Interferons have an important role during acute HBV infection as the infected hepatocytes begin production of IFN-α/β that inhibits viral packaging (26, 27). Despite transient IFN production, HBV is called a “stealth” virus as it effectively evades the innate immune response, which leads to undetected infection for months. Chronic infection establishes the immune tolerant phase that involves an asymptomatic patient [without altered serum alanine transferase (ALT) levels or hepatic damage] with HBV, HBsAg, and HBeAg detectable in serum, especially in patients infected perinatally (11). NK cells have been suggested to have a role in acute HBV although there is conflicting evidence about their contribution depending on the stage of pathogenesis of the patients (28). In patients with acute HBV infection, there is reduced activation of NK cells that exhibit decreased cytolytic activity that coincided with peak viremia (29). Furthermore, chronic HBV patients showed decreased NK cell activation markers and reduced IFN-γ and TNF-α production (30). Interestingly, NKT cells, a group of immune cells that share properties with NK cells and signal through T cell receptor (TCR) to lipids, were activated early during HBV infection by lipids generated due to HBV infection and contributed in priming T and B cells (31). Moreover, NKT cells have been shown to cause suppression of HBV replication, albeit in a transgenic HBV mouse model by injection of NKT activating ligand (32).

Kupffer cells, tissue resident macrophages within the sinusoidal compartment of liver, act as first line of defense against pathogens invading the liver. HBV is known to infect hepatocytes in the liver but no direct infection of Kupffer cells has been observed although uptake of HBsAg by these cells has been reported (33). *In vitro* cultures of non-parenchymal cells (composed mostly of Kupffer cells) with HBV or HBsAg showed an increase in IL-6 production that inhibited HBV replication and transcription in HBV-infected PHHs (33, 34). This is in contrast to production of TGF-β and absence of inflammatory cytokines by rat Kupffer cells in response to HBV (35). Since Kupffer cells are tolerogenic in nature, the high expression of PD-L1 and production of anti-inflammatory mediators suggests that these cells might be responsible for reduced T cell activity (35, 36). Dendritic cells (DCs) circulating through the liver also contribute to the immune response against HBV as they are important for priming the adaptive immune response. It was observed that patients with chronic HBV have impaired cytokine production of myeloid DCs (mDCs) and plasmacytoid DCs (pDCs) *in vitro* as compared to healthy control patients (37). Additionally, the number of mDCs in peripheral blood increased in response to adefovir drug treatment for chronic HBV (38). mDCs were also found to have elevated expression of PD-L1 in chronic HBV patients that resulted in reduced T cell activation *in vitro* (39). However, the role of these innate immune cells is still not very clear as most of these studies look at peripheral blood DC populations and do not specifically define the role of hepatic DC function for HBV infection. Additional studies are required to clearly outline the function of these cell types in HBV pathogenesis and modify HBV treatment strategies for better immune response.

Cell-mediated immunity is critical for clearance of HBV infection from hepatocytes. CD8^+^ and CD4^+^ T cells have been shown to be indispensable for resolution of HBV infection in infected chimpanzees (40). Interestingly, the mechanism for this clearance was non-cytolytic for hepatocytes and occurred through interferon-α and TNF-α provided by CD8^+^ and CD4^+^ cells when infected with high viral inoculum (41). Furthermore, it was observed that quality of immune response to HBV and clearance of infection was dependent on the viral inoculum size and CD4^+^ T cell priming (42). CD4^+^ T cell depletion prior to infection or early on during HBV infection resulted in loss of CD8^+^-dependent T-cell response whereas CD8^+^ T cell depletion during acute HBV resulted in failure of HBV clearance (42). However, during the later stages of acute infection, this mechanism was observed to be IFN-γ-dependent CD8^+^-mediated cytolytic mechanism with considerable injury to infected hepatocytes as seen in HBV-infected chimpanzees (43). The repertoire of CD8^+^ epitopes during acute infection is diverse for Pol, envelope, and nucleocapsid antigens that changes to less functional envelope and core-specific T cells with increased detection of functional Pol-specific T cells during chronic phase of infection (44, 45). CD4^+^ T cells follow the same pattern of epitope recognition, but are most responsive to core proteins (44). B cell response plays an integral role for HBV detection and resolution where antibody response is very critical in different phases of HBV infection as antibody titers are used to categorize the extent of disease. Antibodies are produced early on against all HBV proteins in an acute infection but anti-HBsAg provides protective immunity against subsequent infections (46, 47).

Chronic HBV infection is characterized by the persistence of HBsAg, HBeAg, and HBV DNA for more than 6 months (1). Chronic HBV carriers can remain asymptomatic but a small percentage of patients can undergo viral re-activation and develop hepatocellular carcinoma (HCC) (48). Moreover, pregnant mothers who are HBeAg positive are at high risk of transmitting HBV infection to their infants, who develop chronic HBV (48). These infants undergo the immune tolerant phase of chronic HBV infection with presence of HBV DNA in their blood but have normal liver function and absence of hepatic inflammation (11, 49). This can be followed by conversion to immune active phase with increased ALT levels, detection of HBeAg, and increased hepatic inflammation and fibrosis at later stages (49). An important marker for determining the fate of chronic HBV infection is the seroconversion to anti-HBeAg, as loss of HBeAg increases the chances of disease remission (49). The loss of HBeAg occurs coincides with the production of anti-HBeAg antibody in the inactive carrier phase, usually asymptomatic for most patients but some patients can exhibit occasional hepatitis symptoms (48, 50). Although the loss of HBeAg was thought to be an immune escape mechanism by the virus to reduce T cell response against core antigen, there is evidence to suggest that CTL priming is similar for both HBeAg positive and negative strains *in vitro* (51). However, there is more significant *in vivo* damage to liver with expression of HBeAg as opposed to HBcAg alone (51).

T cells can efficiently clear out HBV infection during the acute phase but they become unresponsive due to exhaustion in the chronic phase. T cells express lower levels of CD127 (IL-7R) and higher levels of PD-1 and CTLA4 during chronic HBV infection (52, 53). Lower levels of PD-1 coincided with high T-bet levels, a transcription factor that negatively regulates PD-1 expression, in T cells from chronic HBV patients (54). Similarly, another inhibitory costimulatory molecule, 2B4, was found to be upregulated along with PD-1 in liver and blood of HBV-infected patients (55). Therefore, it is postulated that an increase in expression of inhibitory cell surface receptors causes T cell exhaustion in chronic HBV as has been observed in other chronic viral infections and causes decreased functional response to infected hepatocytes.

## Model Systems for HBV

Since HBV replication and infection is hepatotropic, the most commonly used model systems are hepatocyte cell lines derived from HCC patients. These cell lines (Huh7, HepAD38, HepG2) are useful in studying the viral replication *in vitro* and are convenient to grow in culture. Huh7 and HepG2 cannot be infected by HBV but they support HBV replication once HBV genome is transfected into these cells (56, 57). A drawback of these cell lines is the inability to investigate HBV entry using these as model systems, which can be overcome with overexpression of NTCP (58). HepaRG is a cell line derived from chronic hepatitis C tumor that contains hepatic progenitor cells and can be naturally infected with HBV after differentiation by addition of dimethyl sulfoxide (59). Even though these cells allow for HBV infection and cccDNA formation, these cells require a complex differentiation regime to be able to polarize and permissive to infection. Despite the ability to infect these cells, the relative infection rates are still quite low and cccDNA amplification does not occur (60). HepAD38 cell line allows for HBV replication under the control of tetracycline induction (61). However, these cell lines are transformed and lack innate cytoplasmic sensors, which makes deciphering the cellular response to HBV infection complicated. PHHs can be used for HBV infection but they have limited replication ability in tissue culture and are refractory to genetic manipulation (62). Furthermore, PHH availability from human liver surgical resections can be limited and variable in genetic composition that can affect HBV infectivity (63).

Another challenge in the study of HBV is the limited number of animal species that can be naturally infected with HBV. The animal model system that most closely resembles human HBV infection is chimpanzees. Chimpanzees have HBV kinetics similar to humans and can undergo both acute and chronic infection (64). The immune response to HBV infection has been studied in this model system and contributes greatly to our current understanding of HBV pathogenesis. However, the high costs associated with purchasing chimpanzees and the restrictions imposed on use of primates as models for biomedical research limit the use of chimpanzees for HBV research. Additionally, chimpanzees have milder disease pathology for HBV as compared to humans, less antibody response, and rare vertical transmission, which are important modes of HBV transmission in humans (9, 65). Tupaia (tree shrew) can also be infected with HBV and was used as a model system to study HBV replication, acute, and chronic HBV infection (7, 66, 67). The other model systems commonly used are duck HBV and woodchuck hepatitis virus. Both these systems can develop chronic HBV and model HBV pathogenesis, cccDNA formation, HCC, and response to drugs very well (68–74). However, the breeding of woodchucks and lack of detailed knowledge of their immune system makes it harder to use these animals in lab (69). Moreover, duck HBV belongs to a different genus, *Avihepadnaviridae*, of viruses that is orthologous to *Orthohepadna* and lacks the fourth open reading frame that encodes HBx protein that hinders investigating the role of HBx which remains an unanswered question in the field (71, 75).

Mice are the most favored animal model system chosen to study immune responses to viruses. However, mice cannot be naturally infected with HBV virus. Mouse hepatocytes are not permissive for HBV entry and are unable to support HBV replication and cccDNA formation (14). Conversely, an immortalized mouse cell line has been shown to sustain rcDNA formation under inducible conditions for 1 week (76). HBV transgenic mice that express HBV antigens (core, envelope, surface) in the liver have been established by different groups (77–79). This important development permits investigation of the immune response against these specific antigens. However, these transgenic models are limited by the fact that they are tolerized to HBV antigens they express and do not allow studies of viral replication. An important milestone was the generation of transgenic mice with severe combined immunodeficiency that are not tolerized to HBV antigens and allowed for development of chronic liver disease similar to humans (80). Another model system utilizes hydrodynamic injection of HBV genome encoded on an adeno-associated virus plasmid which results in the development of both acute and chronic infection in C57BL/6 mice (81). Low doses administration of adenovirus vector caused persistence of HBV infection without an adaptive response and allowed studies of chronic HBV infection (82). Recently, human chimeric mice have been developed that allow human hepatocytes to regenerate mouse liver and allow HBV infection to persist (83, 84). These mice can further be humanized by expression of human MHC molecules to recapitulate the human disease (85). However, the biggest drawbacks of mice as model systems are the artificial system for establishing HBV infection and the lack of cccDNA establishment in mouse hepatocytes (summarized in Table 1).

**Table 1:** **Model systems for hepatitis B virus (HBV)**.

Model system	Uses and advantages	Disadvantages	Reference
Cell lines (HepG2, Huh7 HepAD38, HepaRG)	HBV replication and transcription Drug metabolism Cultured indefinitely	Transformed and dysfunctional innate responses Lack of liver architecture	(56, 57, 59, 61, 62, 76)

Primary human hepatocytes	HBV replication and transcription Drug metabolism Innate immune responses	Cannot be cultured indefinitely Lack of liver architecture	(62)

Duck	Viral life cycle Drug metabolism Chronic infection and cccDNA formation	Lack of research tools Ortholog of HBV	(68, 71, 72)

Tupaia (tree shrew)	Experimental infection of HBV and viral lifecycle Chronic infection and cccDNA formation	Lack of research tools	(7, 66, 67)

Woodchuck	Experimental infection of HBV and viral lifecycle Chronic infection and cccDNA formation Drug metabolism Liver disease and carcinogenesis	Lack of research tools Ortholog of HBV	(69, 70, 74)

Chimpanzee	Experimental infection of HBV and viral lifecycle Immune response to HBV Vaccine research Drug metabolism	Less severe disease Ethical considerations to use of primates in research	(40–43, 65, 89, 105)

Mouse	Immunotherapy Drug metabolism	No experimental HBV infection	(77, 79, 81, 83–85)

Despite the current model systems, there is a paucity of animal models that can fully recapitulate HBV replication and lifecycle. Additionally, there is lack of knowledge about development of chronic HBV infections in humans and the establishment of cccDNA, a key step in HBV infection, which is not supported by most systems or occurs at very low levels. The challenge of finding an effective therapeutic approach partly lies with inability to model different aspects of HBV lifecycle.

## Therapies for HBV

Conventional therapies for HBV include use of nucleoside/nucleotide reverse transcriptase inhibitors (NRTIs or NtRTIs) that are commonly used as antivirals which target the reverse transcription that is critical to the life cycle of HBV (86). Lamivudine, a cytidine analog and the first effective NRTI against HBV, resulted in reduced ALT levels, decreased serum HBV DNA levels, and HBeAg seroconversion (86). However, the biggest drawback of prolonged use of this drug was the occurrence of drug-resistant mutants (87). Other RTIs introduced were adefovir, an adenosine monophosphate, and entecavir, a guanosine analog, which had increased efficacy at reducing HBV DNA levels and ALT levels (88, 89). Adefovir developed similar drug-resistance problems as lamivudine and also showed nephrotoxicity at higher dosage (90, 91). Entecavir was more refractory to development of drug-resistance and exhibited pre-disposition to drug-resistant mutants for the mutants with lamivudine resistance (92). Tenofovir is the most recent NtRTI that competes with the adenosine and acts as a chain terminator during replication using HBV Pol (93). There is no documentation of tenofovir resistance but it is nephrotoxic over prolonged usage (94).

Cytokine-mediated therapy includes polyethylene glycol linked interferon alpha (PEG-IFN-α) treatment to boost immune system and production of interferon-stimulated genes (ISGs) (95). IFN-α treatment is recommended for patients with chronic HBV infection and no liver disease history because the adverse effects of IFN-α include flu-like symptoms and hepatic flares (96). IFN-α treatment results in lower viral replication and reduction in serum HBV DNA as IFN-α causes reduced HBV transcription and enhancer activity (97). Chronic HBV patients administered subcutaneous or intramuscular IFN-α resulted in reduction of HBV virions as well as DNA Pol and HBcAg linked to HBV virions (98). Other than interfering with HBV replication at various steps of HBV lifecycle, IFN-α was shown to upregulate APOBEC3A and APOBEC3B that caused cytidine deamination and degradation of cccDNA without influencing the host genome (99). Similar to better efficacy for HCV treatment with PEG-IFN-α, the use of PEG-IFN-α compared to standard IFN-α resulted in a significant reduction of HBV DNA and ALT levels in chronic HBV patients (100). Recently, toll-like receptor 7 (TLR-7) agonists have been utilized in chronically infected chimpanzees to stimulate IFN-α as well as ISGs and reduce HBV DNA in serum (101). TLR-7 is induced by single-stranded RNA in endosomes, induces expression of IFN-α and pro-inflammatory cytokines and is highly expressed by pDCs that are potent type-I IFN-producing cells. This makes TLR-7 a suitable target to drive an enhanced interferon response and aid in HBV disease amelioration. In a woodchuck model of woodchuck hepatitis virus, the use of TLR-7 agonist GS-9260 resulted in lower viremia and lower rate of HCC for chronically infected woodchucks (102). However, TLR-7 agonist GS-9620 response in chronic HBV patients resulted in a transient increase in interferon-stimulated gene 15 but no change in IFN-α within 2 days of agonist administration (103). Another report for use of GS-9260 in chronic HBV patients reported the activation of ISGs for low and high doses of the drug but IFN-α was detected only at high doses that also resulted in increase in adverse events for the patients. Thus, the use of an oral TLR-7 agonist may be beneficial but its efficacy as a combinatorial therapy for chronic HBV still remains to be investigated.

For antibody response, vaccines for HBsAg as well as DNA-based vaccines were developed against HBV and there are various vaccination strategies currently being evaluated in clinical trials ranging from DNA-based vaccines to HBV recombinant proteins (104). HBsAg vaccine is prophylactic in nature and effective in generating anti-HBsAg antibodies for long-term immunity (47, 105). A DNA-based vaccine utilizing a plasmid-encoded HBV that was administered intra-muscularly to chronic HBV patients, generated a transient T-cell response against HBV, but did not result in long-term clearance of HBV (106). HBsAg in combination with adjuvant immune complexes were administered to a group of chronic HBV patients to stimulate DC response and a modest response of late HBeAg seroconversion was observed (107). Other approaches included the combinatorial use of antiviral drugs or cytokines with vaccination to achieve better immunity. The T cell response with combined use of lamivudine and intradermal HBsAg vaccination was enhanced and lead to effective seroconversion in chronic HBV patients (108). A similar strategy utilized multiple plasmids encoding for HBV genes and modified human interleukin 12 gene for intramuscular vaccination and reported lowered HBV DNA levels following treatment (109). However, drug-based treatment may inhibit viral replication but does not allow complete removal of cccDNA, which can persist with minimal transcription. Other HBV therapy strategies involve targeting different stages of the viral lifecycle. Myrcludex B is a NTCP inhibitor that is currently in clinical trials to prevent HBV entry into hepatocytes (110). The next important target in the HBV lifecycle is core or capsid protein as it is critical for viral assembly. Drugs that target capsid protein or disrupt viral assembly (NVR1221/3778: Novira Therapeuticals, Bay41-4109: AiCuris) have been developed and are being evaluated in pre-clinical trials (111). Another focus for therapy is HBsAg targeted by REP 2139-Ca, a nucleic acid polymer from Replicor Inc. that inhibits release of HBsAg that causes suppression of the immune response. Once the release of HBsAg is blocked, the immune system can respond and clear out the HBV infection. Interfering RNA or siNRA (ARC 521: Arrowhead Pharmaceuticals, TKLM HBV: Tekmira, ALN HBV: Alnylam) and anti-sense RNA (ISIS HBV: Isis) have been developed by various pharmaceutical companies to target viral mRNAs from both cccDNA as well as integrated HBV genome with encouraging preliminary results that are currently in pre-clinical/clinical trials for chronic HBV patients. Other strategies to inhibit HBV include use of innate nucleic acid sensor agonists to increase IFN-α production or targeting cccDNA transcriptional template of HBV. HBV cccDNA exists as an episome and can undergo epigenetic modifications aided by HBx protein (112). This ability of cccDNA to undergo post-translation modifications paves way for developing therapeutics that can be targeted to inhibit post-translation modifications of cccDNA. Interestingly, these epigenetic modifications of cccDNA are responsive to IFN-α that causes reduction of transcription-activating histone acetylation and methylation on cccDNA (113, 114). Thus, use of epigenetic inhibitors targeted to infected cells or modulation of IFN-α could be effective in removal of cccDNA from HBV-infected cells. Genome editing tools such as CRISPR and small molecule drugs have been used to specifically target HBV cccDNA within hepatocytes *in vivo* whereas HBV core protein inhibitors are being screened for potential inhibitors to viral assembly (115–117). Importantly, none of these therapies have resulted in complete clearance of HBV in chronic HBV patients or resulted in long-term recovery of patients.

## Concluding Remarks and Future Challenges

**Figure 1:**
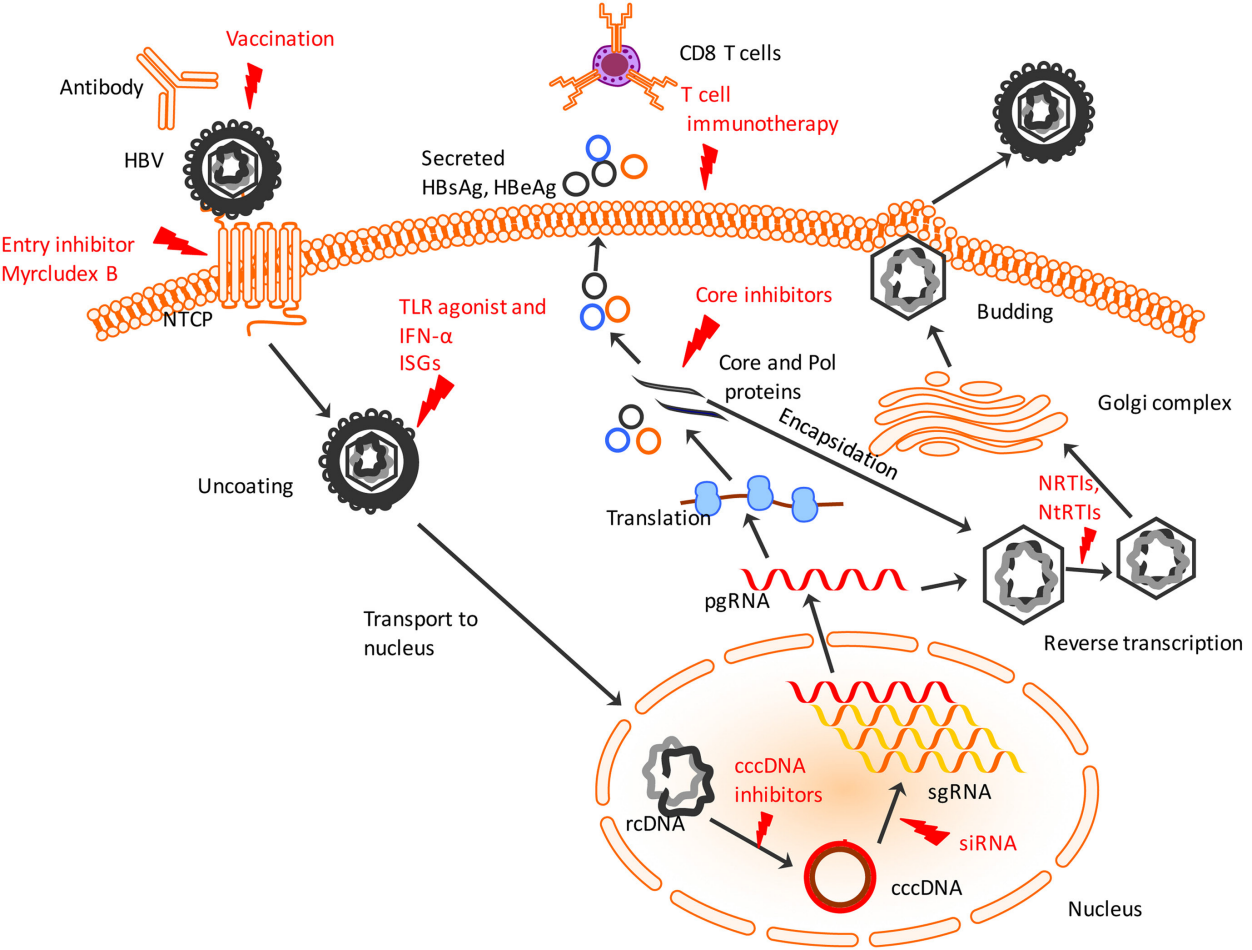
**Hepatitis B virus (HBV) lifecycle and different steps of the viral lifecycle that can be targeted for therapy**. HBV enters using sodium taurocholate cotransporting polypeptide (NTCP) receptor which entry inhibitors like Myrcludex B can target to prevent HBV entry. The virus uncoats and relaxed, circular DNA (rcDNA) within the nucleocapsid is shuttled to nucleus. IFN-α and TLR agonists work to induce ISGs to induce an antiviral state within infected and uninfected hepatocytes. The conversion of the rcDNA to cccDNA is a key step for establishing chronicity of HBV infection which can be targeted by cccDNA drug inhibitors or genome editing tools. Post-transcription of pre-genomic RNA (pgRNA) and sub-genomic RNA (sgRNA), the pgRNA gets translated into HBV proteins including core and polymerase (Pol) proteins that undergo encapsidation to form nucleocapsid. Small drug-like molecules target the core protein to inhibit viral nucleocapsid assembly. Within the nucleocapsid, reverse transcription occurs that is targeted by NRTIs and NtRTIs. Subsequently, the nucleocapsids acquire a lipid envelope after trafficking through Golgi complex and are released from the hepatocyte surface. Chimeric T cell receptor, adoptive transfer of activated HBV-specific T cells and use of antibodies targeting T cell inhibitory proteins activates cytotoxic T lymphocytes to recognize and lyse infected hepatocytes. Red indicates current or potential therapeutic targets. TLR, toll-like receptor; ISGs, interferon-stimulated genes; NRTIs, nucleoside reverse transcriptase inhibitors; NtRTIs, nucleotide reverse transcriptase inhibitors.

The newer generation of treatment strategies involves the use of cell-based therapies utilizing T-cells for priming the innate and adaptive effectors. Since T cells are the mediators of HBV clearance during recovery in an acute infection, it would be an effective strategy to trigger or enhance their response and resolve chronic HBV infection. Most importantly, targeting-infected hepatocytes may result in clearance of cccDNA as well. Several groups have utilized adoptive transfer of memory T cells specific to HBV to generate an immune response as opposed to relying only on the exhausted T cells found in chronic infection. HBV-specific TCR was introduced into human hepatocytes from acute as well as chronic patients (46). These genetically modified TCRs recognized HBV antigens, generated a functional response against HBV-infected cells by producing cytokines (IFN-γ, TNF-α) and lysed the cells *in vitro* and *in vivo* in mice containing HepG2-derived tumors (118). The use of chimeric TCR with the antigen recognition domain derived from the antibodies against HBsAg and linked to T-cell receptor signaling was effective in generating cytolytic response against HBV-infected hepatocytes and eliminating cccDNA *in vitro* (119). Furthermore, utilizing strategies to block inhibitory receptors on T cells in chronic HBV patients should boost the ability of these T cells to functionally respond to infected hepatocytes. Blockage of inhibitory receptors PD-1 on T cells has been shown to be effective in improving the immune response by increased IFN-γ production and induced HBV clearance in mouse model of HBV (120). Similarly, blocking 2B4 and CTLA4 increased antiviral T cell proliferation and cytotoxicity (42, 53, 55). Thus, reversing the depletion of T cells in HBV and resuscitating their effector function could prove to be an effective approach against this disease. These different strategies can augment the immune system and provide moderate intervention. The biggest therapeutic challenge in HBV, however, is overcoming the highly resistant cccDNA which reside in nuclei of infected hepatocytes and can evade detection by the immune response (summarized in Figure 1). Therefore, future treatments need to target the cccDNA to effectively clear HBV and prevent HCC due to HBV chronic infection.

## Author Contributions

JS and PS conceived and wrote the manuscript. MH, TH-B, and SR critically revised and approved the final version of the manuscript.

## Conflict of Interest Statement

The authors declare that the research was conducted in the absence of any commercial or financial relationships that could be construed as a potential conflict of interest.
